# Sphingomyelinase Activity of *Trichomonas vaginalis* Extract and Subfractions

**DOI:** 10.1155/2013/679365

**Published:** 2013-08-19

**Authors:** Francisco González-Salazar, Jesús N. Garza-González, Carlos E. Hernandez-Luna, Benito David Mata-Cárdenas, Pilar Carranza-Rosales, Jorge Enrique Castro-Garza, Magda Elizabeth Hernández-García, Javier Vargas-Villarreal

**Affiliations:** ^1^División de Biología Celular y Molecular, Centro de Investigación Biomédica del Noreste, Instituto Mexicano del Seguro Social, Administración de Correo No. 4, 2 de abril 501 Colonia Independencia, 64720 Monterrey, NL, Mexico; ^2^Departamento de Ciencias Básicas, División de Ciencias de la Salud, Universidad de Monterrey, Avenida Morones Prieto 4500 Pte, 66238 San Pedro Garza García, NL, Mexico; ^3^Departamento de Biología Celular y Genética, Facultad de Ciencias Biológicas, Universidad Autónoma de Nuevo León, 66451 San Nicolás de los Garza, NL, Mexico; ^4^Laboratorio de Enzimología, Facultad de Ciencias Biológicas, UANL, San Nicolás de los Garza, NL, Mexico; ^5^Facultad de Ciencias Químicas, Universidad Autónoma de Nuevo León, Avenida Manuel L Barragán S/N, San Nicolás de los Garza, NL, Mexico

## Abstract

Trichomoniasis is one of the most common acute sexually transmitted curable diseases, and it is disseminated worldwide generating more than 170 million cases annually. *Trichomonas vaginalis* is the parasite that causes trichomoniasis and has the ability to destroy cell monolayers of the vaginal mucosa *in vitro*. Sphingomyelinases (SMase) are enzymes that catalyze the hydrolysis of sphingomyelin into ceramide and phosphorylcholine. Ceramide appears to be a second messenger lipid in programmed apoptosis, cell differentiation, and cell proliferation. Sphingomyelinase is probably a major source of ceramide in cells. Signal transduction mediated by ceramide leads cells to produce cytokine induced apoptosis during several inflammatory responses. SMase are also relevant toxins in several microorganisms. The main objective of this research is to identify SMase activity of *T. vaginalis* in the total extract (TE), P30, and S30 subfractions from brooked trophozoites. It was found that these fractions of *T. vaginalis* have SMase activity, which comes principally from P30 subfraction and was mainly type C. Enzymatic activity of SMase increased linearly with time and is pH dependent with two peaks by pH 5.5 and pH 7.5. The addition of manganese to the reaction mixture increased the SMase activity by 1.97.

## 1. Introduction

Trichomoniasis is caused by the unicellular flagellated protozoan parasite named *Trichomonas vaginalis* which is one of the most prevalent sexually transmitted diseases. It has a worldwide distribution, and WHO estimates that more than 170 million cases are reported each year [1]; of these, 18.5 million come from Latin America [2]. In Mexico, more than 125,000 new cases are reported annually [3]. *T. vaginalis* infects both genders. In men this infection is commonly asymptomatic; however it may cause urethritis, prostatitis, cystitis, epididymitis, and infertility. In women the infection normally causes symptoms of vulvovaginitis and urethritis with vaginal discharge, irritation, dysuria, and abdominal pain. Vaginal secretion may also be yellow-green, itchy, frothy, and foul-smelling [4]. In pregnant women this disease has been related to premature rupture of amniotic membranes, premature birth, and low birth weight [5]. Patients with trichomoniasis are more susceptible to develop HIV seroconversion [6]. 

*T. vaginalis* is pyriform and grows in microaerophilic conditions when cultured. It has two main stages: flagellated and trophozoite [7]. To this date, there is no knowledge of resistant cysts forms [4]. *T. vaginalis* does not have mitochondria, instead it has hydrogenosomes, organelles with no DNA, formed by three chromatic granules [8]. Energy requirements are provided by the transformation of glucose to glycerol and succinate in the cytoplasm, followed by the subsequent conversion of malate to pyruvate, hydrogen, and acetate in the hydrogenosomes [9–11]. *T. vaginalis* has the ability to destroy monolayers of epithelial cells isolated from human vaginal mucosa *in vitro* by detaching them, lysing them [12–14], or by phagocytosis [15, 16]. Engbring and Alderete [17] reported that *T. vaginalis* has a high specificity to bind only to mucosal epithelial cells of the genitourinary tract. This process is mediated by proteases found in the parasite's surface. Some authors have identified and characterized several cysteine proteinases and adhesins that participate in adhesion and cytotoxicity of the parasite to the vagina and ectocervix [18, 19].

Although the pathogenic mechanisms of *T. vaginalis* are unknown, there are some factors related to its destructive effect, also its ability to proliferate and damage host cells [20, 21]. At this time, several parasite molecules have been identified as the cause of damage in cells and tissues of the host [20, 21]. Several hydrolases have been described in *T. vaginalis*; those with low molecular weight may be released into the cell medium [20]. Some of these molecules participate in specific cell damage including neuraminidase [22], *β*-N-acetylglucosaminidase (EC 3.2.1.30), *α*-mannosidase (EC 3.2.1.24), *β*-glucosidase (EC 3.2.1.21), acid phosphatase (EC 3.1.3.2), cysteine proteases [17, 20, 23], and phospholipases [21]. Also, an additional molecule of membrane attack has been recently detected in *T. vaginalis* and called lytic factor, which is able to destroy cells and nucleate erythrocytes as well as acting specifically on phosphatidylcholine suggesting an activity of phospholipase A^2^. Vargas-Villarreal et al. [21] demonstrated direct and indirect activities dependent of hemolytic phospholipase A (A^1^ and A^2^) in subcellular extracts from *T. vaginalis*. These activities have been proposed as responsible for hemolytic and cytolytic effects of *T. vaginalis*.

Sphingomyelinases (SMase) are enzymes that catalyze the hydrolysis of sphingomyelin into ceramide and phosphorylcholine and are defined as EC 3.1.4.12. They are classified according to their pH requirements (alkaline, neutral, and acidic) and of these enzymes the most common are the SMase type C (SMase-C, EC 3.1.4.12) and SMase type D (SMase-D, EC 3.1.4.41). Both enzymes degrade sphingomyelin, but SMAase C produces sphingomyelinase and ceramide phosphorylcholine while SMAase D produce choline and phosphorylceramide [24].

Enzymes with sphingomyelinase activity have been identified and proposed as possible virulence factors in other organisms such as the violin spider venom [25], *Clostridium perfringens* [26], *Bacillus cereus* [23], *Staphylococcus aureus* [23], *Leptospira interrogans* [27], and *Neisseria gonorrhoeae* [28]. This activity is important in physiological and pathophysiological processes in mammalian cells such as sphingomyelin digestion in lysosomes [29]. Ceramides are involved as second messengers in cytokine inducing apoptosis [30–32], cell differentiation [33], and in the immune and inflammatory responses [34]. 

 The main objective of this research was to identify sphingomyelinase activity in the total extract, P30, and S30 subfractions of *T. vaginalis*.

## 2. Material and Methods

### 2.1. Biological Material

#### 2.1.1. *Trichomonas vaginalis* Strain

Strain GT-15 of *T. vaginalis* was gently donated to us by Dr. Fernando Anaya-Velázquez from the Experimental Biology Institute, Chemistry Faculty, Guanajuato University, Mexico. It was maintained under cryopreservation and reseeds three times per week in our laboratory in PEPHS medium, supplemented with 10% (v/v) bovine serum and Diamond's vitamins-Tween 80 mixture [35]. The strain of *T. vaginalis* remains in three tubes at a time. The best growth culture was inoculated 5 × 10^3^ trophozoites/mL in three new tubes with fresh PEHPS [7, 36, 37]. Trophozoites used in the experiments were grown in suspension in spinner flasks [7, 21, 38].

### 2.2. Preparation of Subcellular Fractions

The subcellular fractions were prepared as described previously [38]. Briefly, pellet containing trophozoites harvested from the spinner flasks were suspended in two volumes of Hank's balanced salt solution BSS (0.7 mM CaCl_2_, 5.5 mM Glucose, 120 mM NaCl, 5.3 mM KCl, 1.7 mM MgSo_4_, 1 mM Trizma base, and pH 7.5). The trophozoites were disrupted with an electric motor-driven Potter-Elvehjem Teflon-glass homogenizer (Bellco, Glass Inc., NY, USA) [38] and activated at 1000 rpm, representing the fraction total extract (TE). This fraction was separated in two parts; the first 3 mL of extract was divided in 0.5 mL aliquots and stored at −70°C until required. The remaining TE was centrifuged at 30,000 ×g during 15 min at 4°C. The resultant supernatant (S30) was stored until being used. The pellet (P30) was resuspended with 1 volume BSS, distributed in 200 *μ*L aliquots, and stored at −70°C. Before the initiation of each experiment, a sufficient numbers of TE, P30, and S30 aliquots were thawed at room temperature and diluted with BSS to adjust the proteins concentration, according to each experiment design.

### 2.3. Determining Sphingomyelinase Activity

Sphingomyelinase activity (SMAase) was determined by radio assay in soluble and particulate samples. It was previously described by Vargas-Villarreal et al. [38]. Briefly, substrate was prepared by mixing 1 mL of 100 mM Trizma base (pH 7.5) solution, 1 mM MgCl_2_, 0.2% of Tritón X 100, 4 mg sphingomyelin, and 2.5 *μ*Ci [N-methyl-^14^C]-sphingomyelin ([^14^C]-sphingomyelin [47 mCi/mmol]) (PerkinElmer Life and Analytical Science, Boston, MA, USA), in 1.5 mL borosilicate (Bellco, Glass Inc., Vineland, NJ, USA). The mixture was sonicated in an Ultratip Labsonic System (Lab-Line Instrument Inc., Melrose Park, IL, USA), applying one pulse of 40 W for 60 s. This substrate preparation was divided into 0.5 mL aliquots and stored in vials at −70°C until being used. 

A 10 *μ*L of assay mixture and 10 *μ*L 2X mixture containing several amounts of fractions from *T. vaginalis*, TE, P30, or S30 (0–400 *μ*g of total protein of each fraction), were deposited in tubes 7 × 75 mm borosilicate (Bellco, Glass Inc., NY, USA). Tubes were shaken on vortex for 30 s and incubated at 37°C for 150 min in a moist chamber. After incubation time, the reaction was stopped by adding 25 *μ*L of 1 mg/mL sphingomyelin, 1 mg/mL phosphorylcholine, and 1 mg/mL choline (Sigma Chemical Co, St Louis, MO, USA) in 5% trichloroacetic acid in n-butanol. Then the lipids from each sample containing nondigested sphingomyelin were separated from the SMase hydrolysis products by thin layer chromatography (TLC) [38]. 

### 2.4. Thin Layer Chromatography

The assay mixtures (45 *μ*L) were applied on 10 × 10 cm silica gel plates (0.25 mm thick, 60-mesh, Merck, Darmstadt, Germany). The plates were placed separated into a TLC developing tank containing a mobile phase chloroform/methanol/water (65 : 25 : 4, v/v). Spots corresponding to choline, phosphorylcholine (origin), and sphingomyelin (*R*_*f*_ = 0.29) were developed by exposing the TLC plates to iodine vapors for 10 min [39]. 

To identify the [^14^C]-sphingomyelin, [^14^C]-phosphorylcholine, and [^14^C]-choline spots, their respective relative migration coefficients (*R*_*f*_) were compared with those of their corresponding nonradioactive standards (Sigma). Visualization of spots corresponding to [^14^C]-sphingomyelin, [^14^C]-phosphorylcholine, and [^14^C]-choline was scraped from the TLC silica gel plates and placed into plastic vials containing 5 mL scintillation liquid (BCS, Biodegradable Counting Scintillation fluid; Amersham). Radioactivity in each vial was determined with a 1600 Tri-Carb liquid scintillation spectrometer (Packard Instrument Company, Inc., Downers Grover, IL, USA). The instrument was adjusted to work with unquenched samples with 96% efficiency. One unit of SMase activity was defined as 1 pmol [^14^C]-sphingomyelin hydrolyzed (equivalent to the number of picomoles of [^14^C]-phosphorylcholine released) in 1 hr of incubation. Specific activity was defined as the amount of units of SMase activity per milligram of total *Trichomonas* proteins for 1 hr incubation at 36.5°C (U SMase/mg/hr). 

The type of SMase activity was classified by its cleavage site. *Trichomonas* preparations (TE, P30, and S30; 400 *μ*g/mL) were assayed (at pH 7.5), using the method of sphingomyelinase chromatography bidirectional thin plate, When using protease inhibitors sphingomyelinase activity increases tenfold. The radioactive hydrolysis products were identified by comparing their final location with those of their respective standard [23]. 

The first mobile phase contained methanol/water (25 : 25 v/v) while chloroform/methanol/water (65 : 25 : 4 v/v) was used as the second mobile phase [38].

All determinations were performed three times in triplicate and were presented as the mean ±1 specific activity of the SMase (SMase U/mg total protein/hr) was arbitrarily defined as 1 U = 1 DPM SMAase.

### 2.5. Effects of Inhibitors, Incubation Time, Dose of Proteins, pH, and Dissolvent Cations on Trichomonas SMase Activity

The effect of inhibitors on SMase activity was measured from TE, P30, or S30 fractions in the presence of sodium salt p-chloromercuribenzoate, a protease inhibitor, in a final concentration of 0.1 mM in all fractions [40, 41]. Incubation time was determined by incubation of TE, P30, or S30 assay samples (each containing 400 *μ*g of total protein) for 0–150 min. Dose response curves were obtained using 0–400 *μ*g of P30 total protein. Finally, the effect of pH was analyzed by adjusting the pH values (2–10) with appropriate concentration of glycine-HCl (pH 2–2.5), sodium acetate (pH 3–6), or Trizma base (pH 7–10). The requirement for divalent cations was analyzed by adding 1 mM or 10 mM of MgCl_2_, MnCl_2_, CoCl_2_, CaCl_2_, HgCl_2_, and ZnSO_4_ or 1 or 10 mM EDTA and 10 *μ*L to the P30 fraction. Activity was determined as described previously.

### 2.6. Total Protein Quantification

The concentration of proteins was calculated in biological samples by the method of Lowry et al. [42].

### 2.7. Statistical Analysis

All the experiments were performed three times in triplicate (*n* = 9). Plots of incubation time and dose were analyzed by linear regression, and the results were compared by ANOVA for data normally distributed. 

## 3. Results

### 3.1. Detection of Sphingomyelinase Activity in Total Extracts (TE), P30, and S30 Fractions of *T. vaginalis*

**Figure 1:**
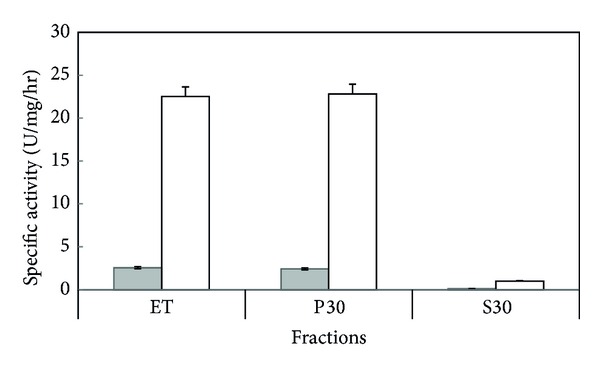
Subcellular distribution of *T. vaginalis* SMase activity. Shadowed and clear bars correspond to specific activity SMase (U SMase/mg/hr) assays with absence or presence of inhibitor p-chloromercuribenzoate, respectively. The total extract (TE) or the subcellular fractions P30 or S30 obtained from *T. vaginalis* trophozoites were evaluated. [^14^C]-sphingomyelin was used as the substrate for all assays. The products of the hydrolysis were [^14^C]-phosphorylcholine or [^14^C]-choline. Each bar represents the mean ± SE from three independent experiments performed in triplicates.

Trichomonas extracts have SMase activity and were able to hydrolyze [^14^C]-sphingomyelin. All fractions (TE, P30, and S30) have this activity. But it was found that sphingomyelinase activity was higher in TE (2.57 U/mg/hr) and P30 (2.43 U/mg/hr), and S30 was less active (Figure 1). When inhibitors proteases as the p-chloromercuribenzoate were used, it was found that sphingomyelinase activity increased in all fractions by a factor of 10 times (Figure 1).

### 3.2. Identification of the Type of Sphingomyelinase-C and an Unidentified ESase Activity Present in TE and P30 of *T. vaginalis*

**Figure 2:**
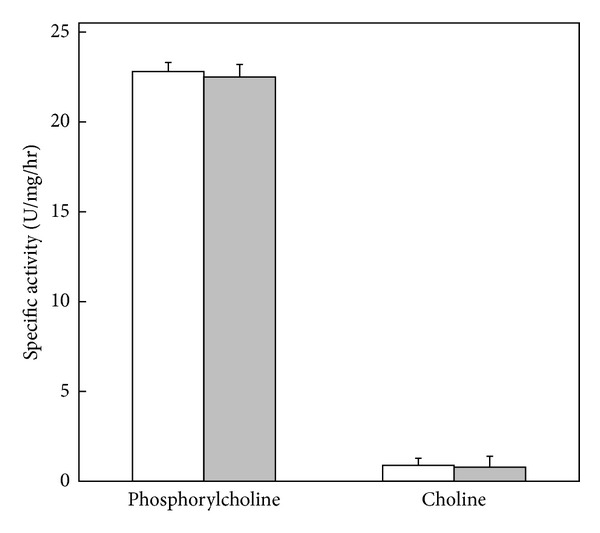
SMase-C activity and unidentified esterase activity in TE and P30 fractions. Shadowed and clear bars correspond to specific activity of SMase-C or esterase activity from TE and P30 fractions from *T. vaginalis* trophozoites, respectively. The hydrolysis products were [^14^C]-phosphorylcholine for SMase-C activity and [^14^C]-choline for esterase activity. Each bar represents the mean ± SE from three independent experiments performed in triplicates.

When sphingomyelinase activity was determined in P30 and TE fractions it was found that virtually all the [^14^C]-phosphorylcholine activities corresponded to 96% and 4% to [^14^C]-choline (Figure 2). It was confirmed that the sphingomyelinase activity of *T. vaginalis* is a type C. In addition, small but reproducible quantities of [^14^C]-choline were detected, indicating the presence of other esterase activity (ESase activity) in TE and P30 fractions (Figure 2). 

### 3.3. Concentration and Time-Dependent SMase-C Activity from P30

**Figure 3:**
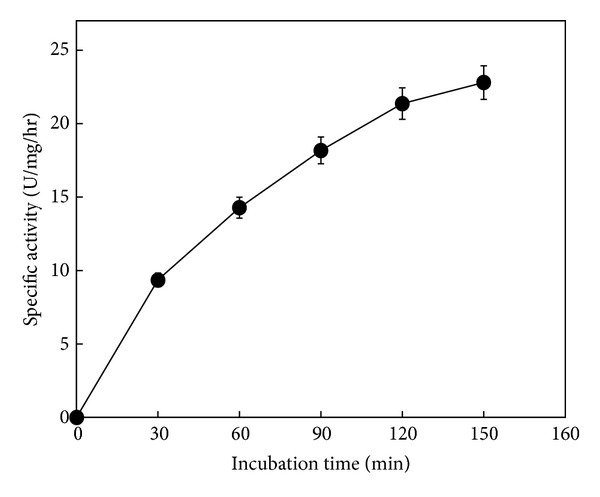
Time-curse of SMase activity from the P30 subcellular fraction. Fractions of P30 containing 400 *μ*g of proteins total by assay were tested by several incubation times (0–150 min). Then [^14^C]-phosphorylcholine released was measured. Symbols correspond to mean ± SE of nine determinations of three independent experiments.

P30 shows a time-dependent SMase C activity; a graphical representation of this activity can be observed in Figure 3. 

**Figure 4:**
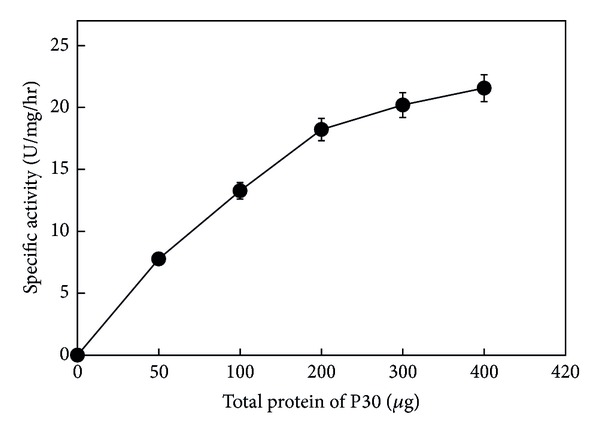
Dose dependence of membrane-associated SMase-C activity. Several protein total concentrations (0–400 *μ*g) of P30 incubated for 150 min at pH 7.5 were tested. Then [^14^C]-phosphorylcholine released was measured. Symbols correspond to mean ± SE of nine determinations of three independent experiments.

 It was found in the dose response curve that the radioactivity in the spots corresponding to [^14^C]-phosphorylcholine steadily increased with increasing concentrations of the P30 fraction from 0 to 400 ug of total protein (Figure 4), showing low proportionality concentrations (less than 100 *μ*g).

### 3.4. Effect of pH on SMase Activity of P30

**Figure 5:**
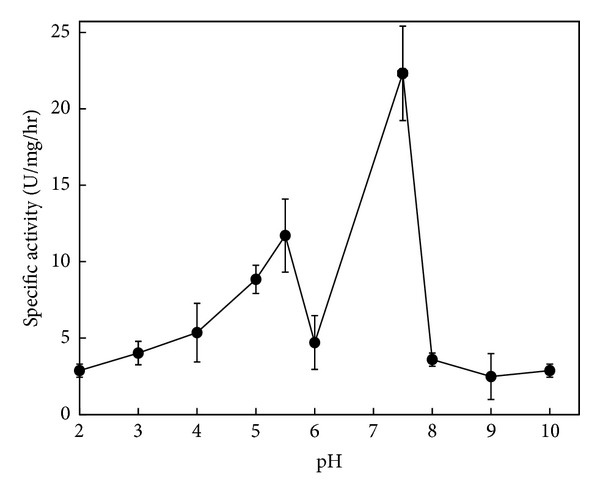
Effect of pH on membrane-associated SMase-C activity. Fractions of P30 containing 400 *μ*g of proteins total by assay were tested by several pH values (2–10). Then [^14^C]-phosphorylcholine released was measured. Symbols correspond to mean ± SE of nine determinations of three independent experiments.

Figure 5 shows that 400 *μ*g of the total protein of the membrane-associated P30 fraction incubated for 150 min at 37°C has two peaks of activity, one at pH 5.5 and the other at pH 7.5. The peak at pH 7.5 corresponds to the highest SMase specific activity and was 1.9 times higher than the acidic activity showed at pH 5. 

### 3.5. Effect of Cations on SMAase Activity of P30

Several cations were tested as described in Table 1; the mixtures were treated with EDTA, MgCl_2_, MnCl_2_, CoCl_2_, CaCl_2_, HgCl_2_, and ZnSO_4_. It was observed that the cation which produced a maximum stimulation effect of Mn^2+^ was 1.97 times more than the control without cations followed by Mg^2+^ and Co^2+^ with results 70 and 84% higher than the control, respectively. Furthermore the effect with EDTA was 0.13 times less than the one which occurred with the control. However, CaCl_2_, HgCl_2_, and ZnSO_4_ cause inhibition of SMase activity by 40 to 93% (see Table 1).

## 4. Discussion

The ability to synthesize toxic substances offers some advantages to several organisms to fend off predators or when capturing a prey. These substances are commonly called poisons and are secreted by glands or buccal organs and in some other cases they are secreted through the skin [43]. Similarly, many microorganisms can produce this type of substances that act as pathogenetic factors favoring the invasion of the host. These substances can cause serious disruption to the host's health [9, 44]. Poisons are usually proteins; the best known are the lipases, phosphatases, hyaluronidases, phospholipases, and sphingomyelinases. Phospholipases and sphingomyelinases are the most studied poisons to date and are recognized to be involved in invasion processes, activation of second messengers, and cytopathogenic mechanisms present in many species of microbes [45].

 We have demonstrated the presence of phospholipases in *Trichomonas* [21], amoeba [46], and giardia [47]. But the presence of sphingomyelinases has not been described in *T. vaginalis.* This study was conducted in order to identify and isolate the production of sphingomyelinase from *T. vaginalis* and thereby build a base of knowledge of the physiopathology of this microorganism that causes serious damage to those affected, such as urethritis, vulvovaginitis, infertility, preterm childbirth, and predisposition to get HIV [6].

*T. vaginalis *is a protozoan with high specificity to bind only to the epithelial cells of the mucosa of the urogenital tract. This process is mediated by proteases found in the parasitic surface and which are decisive in the establishment of infection and participate in pathogenicity. Because once implanted in the vagina the microorganism is able to obtain nutrients from bacteria and leukocytes in the vaginal or urethral cavity, and it is also capable of destroying the host cells [17].

For this to happen, it is necessary first for an invasion to break the integrity of the membranes of the host so the sugar residues present on the surface of the parasite can participate, in particular alpha-D-mannose and N-acetylglucosamine, which are involved in the etching process of *T. vaginalis* [48]. This work suggests that the production of sphingomyelinase helps break the membrane components of the host cell. The *in vitro* cytopathic effect of *T. vaginalis* in MDCK epithelial cells has been intensively studied; these parasite trophozoites produce severe damage to the cell monolayer in 30 minutes and a rapid decrease of the transepithelial resistance [13, 14].

Several researchers have demonstrated virulence factors, proteinases, and adhesins, such as (CP30) which is a 30 kDa proteinase required for parasite adhesion to the target cell [49]: a cysteine proteinase of 65 kDa (CP65) and protein of 120 kDa inducible by high concentrations of iron called AP120 produced by *T. vaginalis* are thought to have cytotoxic activity [50]. Although the pathophysiological mechanisms of *T. vaginalis* are not completely defined, they are now recognized as important virulence factor dependent cell-cell contacts and several secreted factors that cause cell damage as consequence of the symptoms of the patients [20, 21].

GT-15 strain of *T. vaginalis *was selected for being one of the strains that produce higher crop yields [7] and because it was detected and quantified for hemolytic activity of cytolytic phospholipase A in direct and indirect assays in studies realized previously [38].

In this work, the mass culture of *T. vaginalis* was fractionated to identify sphingomyelinase activity present in the total extract (TE) and the subcellular fractions P30 and S30 (Figure 2). The fractions were obtained by mechanical homogenization to preserve as far as possible the subcellular compartmentalization and prevent protein denaturalization caused by freeze-thaw cycles [51, 52]. Bovine serum was not included in the assays to avoid the presence of undefined factors in the reaction mixtures that can interfere with the activity of sphingomyelinase [53].

The activity of sphingomyelinase was determined using the previously developed assay for phospholipase activity and published by Vargas-Villarreal et al. [46], adapted for sphingomyelinase activity detector with modifications to the reaction mixture so as not to exceed 60 *μ*L. This amendment would grant two major advantages to this new method: (a) it is possible to analyze a greater number of samples simultaneously, as it requires fewer radioactivity than other methods [54] and (b) save a considerable amount of reagents.

 Results showed the presence of sphingomyelinase activity in the totality of the extracts of *T. vaginalis* and principally in the P30 fraction. As P30 is a particulate fraction [55], it is likely that such activity is present in the plasma membrane of this protozoan.

To discriminate against that type of activity C or D sphingomyelinase is present in TE and P30 fractions. Was used as substrate [^14^C]-sphingomyelin, whereas if after incubation with P30 and TE the radioactivity was located on the chromatographic spot corresponding to [^14^C]-phosphorylcholine but not in the [^14^C]-choline, then enzymes sphingomyelinase would be of type C. Sphingomyelinase type C activity was confirmed when both fractions showed a remarkable activity in the spots corresponding to [^14^C]-phosphorylcholine, and then these fractions subjected them to a bidirectional chromatography. These results undoubtedly confirm that the sphingomyelinase activity present in *T. vaginalis* is of type C. It was demonstrated that from all of the degradation products of [^14^C]-sphingomyelin 4% were [^14^C]-choline. It corresponds with type D SMase activity, probably caused by unidentified esterase.

In previous studies trichomonas [56] have been shown to have protease activity. Since trichomonas extracts may contain proteases, a protease inhibitor was used for an activity free of inhibitory effects from these enzymes. An inhibitor of proteases that does not interfere with the activity of sphingomyelinase was used in this assay [40, 41]. The outcome shows an increase more than 10 times in the SMase activity (Figure 1). It was shown that the proteases were affecting sphingomyelinase activity in all fractions. 

The effect of pH on the activity of sphingomyelinase in the fraction P30 presents two peaks, one at pH 5.5 and other at pH 7.5; the latter was almost twice as high (Figure 5). This concludes that P30 has at least two isoforms of sphingomyelinase. Previous studies state that the first sphingomyelinase activity acting on pH 5.5 has as preferred cofactor Zn; the second isoform acting at a basic pH of 7.5 requires Mg as cofactor. For now, we characterize the activity of Mg as a dependent alkaline sphingomyelinase.

It was also demonstrated that other cofactors such as manganese and cobalt can stimulate between 71 to 97% of sphingomyelinase activities at pH 7.5. Besides EDTA, calcium, mercury, and zinc inhibit this activity between 39 and 87% (Table 1).

## 5. Conclusions

The main contribution of this work is the identification of SMase activity in the total extract, P30, and S30 fractions of *T. vaginalis*. This activity is principally of type C and is mainly in the subfraction P30. It showed two peaks of activity at pH 5.5 and 7.5. The activity at pH 7.5 can be increased using cofactors principally Mg.

For the future it is necessary to investigate type D SMase activity to determine the presence of esterase in the extracts and subfractions from *T. vaginalis*, as well as study SMase fraction that is active at pH 5.5.

## Figures and Tables

**Table 1:** Normalized SMAase activity modulated by addition of cations and EDTA into assays.

*Trichomonas vaginalis* GT-15 P30 fraction	
Cations	Normalized specific activity
Any	1
EDTA	0.13
MgCl_2_	1.71
MnCl_2_	1.97
CoCl_2_	1.84
CaCl_2_	0.5
HgCl_2_	0.171
ZnSO_4_	0.609

## References

[1] WHO-World Health Organization 2010 http://whqlibdoc.who.int/hq/2001/WHO_HIV_AIDS_2001.02.pdf.

[2] Gülmezoglu AM, Forna F (2000). Interventions for treating trichomoniasis in women. *Cochrane Database of Systematic Reviews*.

[3] CENAVECE Sistema Nacional de Vigilancia Epidemiológica. Epidemiología. Anuario 2010 Morbilidad Nacional, Secretaria de Salud. http://www.dgepi.salud.gob.mx/anuario/html/anuarios.html.

[4] Sorvillo F, Smith L, Kerndt P, Ash L (2001). *Trichomonas vaginalis*, HIV, and African-Americans. *Emerging Infectious Diseases*.

[5] Sutton M, Sternberg M, Koumans EH, McQuillan G, Berman S, Markowitz L (2007). The prevalence of *Trichomonas vaginalis* infection among reproductive-age women in the United States, 2001-2004. *Clinical Infectious Diseases*.

[6] Cohn SE, Clark RA (2003). Sexually transmitted diseases, HIV, and AIDS in women. *Medical Clinics of North America*.

[7] Mata-Cárdenas BD, Vargas-Villarreal J, Navarro-Marmolejo L, Said-Fernández S (1998). Axenic cultivation of *Trichomonas vaginalis* in a serum-free medium. *Journal of Parasitology*.

[8] Lindmark DG, Muller M (1973). Hydrogenosome, a cytoplasmic organelle of the anaerobic flagellate *Tritrichomonas foetus*, and its role in pyruvate metabolism. *Journal of Biological Chemistry*.

[9] Műller M (1993). The hydrogenosome. *Journal of General Microbiology*.

[10] Műller M, Coombs G, Vickerman K, Sleigh M, Warren A (1998). Enzymes and compartmentation of core energy metabolism of anaerobic protist a especial case in the eucariotic evolution. *Evolutionary Relationships Among Protozoa*.

[11] Müller M (1997). Evolutionary origins of trichomonad hydrogenosomes. *Parasitology Today*.

[12] Heath JP (1981). Behaviour and pathogenicity of *Trichomonas vaginalis* in epithelial cell cultures. A study by light and scanning electron microscopy. *British Journal of Venereal Diseases*.

[13] González-Robles A, Lázaro-Haller A, Espinosa-Castellano M, Anaya-Velásquez F, Martínez-Palomo A (1995). *Trichomonas vaginalis*: ultrastructural bases of cytophatic effect. *Journal of Eukaryotic Microbiology*.

[14] Gilbert RO, Elia G, Beach DH, Klaessig S, Singh BN (2000). Cytopathogenic effect of *Trichomonas vaginalis* on human vaginal epithelial cells cultured in vitro. *Infection and Immunity*.

[15] Rendón-Maldonado JG, Espinosa-Cantellano M, González-Robles A, Martínez-Palomo A (1998). *Trichomonas vaginalis*: in vitro phagocytosis of lactobacilli, vaginal epithelial cells, leukocytes, and erythrocytes. *Experimental Parasitology*.

[16] Demirezen A (2001). Phagocytosis of erythrocytes by *Trichomonas vaginalis*: examination of a cervicovaginal smear. *Diagnostic Cytopathology*.

[17] Engbring JA, Alderete JF (1998). Characterization of *Trichomonas vaginalis* AP33 adhesin and cell surface interactive domains. *Microbiology*.

[18] Mendoza-Lopez MR, Becerril-Garcia C, Fattel-Facenda LV (2000). CP30, a cysteine proteinase involved in *Trichomonas vaginalis* cytoadherence. *Infection and Immunity*.

[19] Tsai C-D, Liu H-W, Tai J-H (2002). Characterization of an iron-responsive promoter in the protozoan pathogen *Trichomonas vaginalis*. *Journal of Biological Chemistry*.

[20] Lockwood BC, North MJ, Coombs GH (1988). The release of hydrolases from *Trichomonas vaginalis* and *Tritrichomonas foetus*. *Molecular and Biochemical Parasitology*.

[21] Vargas-Villarreal J, Mata-Cárdenas BD, Palacios-Corona R (2005). *Trichomonas vaginalis*: identification of soluble and membrane-associated phospholipase A_1_ and A_2_ activities with direct and indirect hemolytic effects. *Journal of Parasitology*.

[22] Padilla-Vaca F, Anaya-Velázquez F (1997). Biochemical properties of a neuraminidase of *Trichomonas vaginalis*. *The Journal of Parasitology*.

[23] Goñi FM, Alonso A (2002). Sphingomyelinases: enzymology and membrane activity. *FEBS Letters*.

[24] Okazaki T, Bell RM, Hannun YA (1989). Sphingomyelin turnover induced by vitamin D3 in HL-60 cells. Role in cell differentiation. *Journal of Biological Chemistry*.

[25] Tambourgi DV, Paixao-Cavalcante D, Goncalves de Andrade RM, de Fernandez-Pedroza MF, Magnol FC, Morgan BP (2005). *Loxoceles* sphingomyelinase induces complement-dependent dermonecrosis, neutrophil infiltration and endogrnous gelatinase expresión. *Journal of Investigative Dermatology*.

[26] Flores-Díaz M, Thelestam M, Clark GC, Titball RW, Alape-Girón A (2004). Effects of *Clostridium perfringens* phospholipase C in mammalian cells. *Anaerobe*.

[27] Lee SH, Kim S, Park SC, Kim MJ (2002). Cytotoxic activities of *Leptospira interrogans* hemolysin SphH as a pore-forming protein on mammalian cells. *Infection and Immunity*.

[28] Meyer TF (1999). Pathogenic neisseriae: complexity of pathogen—host cell interplay. *Clinical Infectious Diseases*.

[29] Garcia AF, Benchimol M, Alderete JF (2005). *Trichomonas vaginalis* polyamine metabolism is linked to host cell adherence and cytotoxicity. *Infection and Immunity*.

[30] Schissel SL, Schuchman EH, Williams KJ, Tabas I (1996). Zn^2+^-stimulated sphingomyelinase is secreted by many cell types and is a product of the acid sphingomyelinase gene. *Journal of Biological Chemistry*.

[31] Rodrigues-Lima F, Fensome AC, Josephs M, Evans J, Veldman RJ, Katan M (2000). Structural requirements for catalysis and membrane targeting of mammalian enzymes with neutral sphingomyelinase and lysophospholipid phospholipase C activities: analysis by chemical modification and site-directed mutagenesis. *Journal of Biological Chemistry*.

[32] Chatterjee S, Han H, Rollins S, Cleveland T (1999). Neutral sphingomyelinase from human urine. Purification and preparation of monospecific antibodies. *Journal of Biological Chemistry*.

[33] Tabas I (1999). Secretory sphingomyelinase. *Chemistry and Physics of Lipids*.

[34] Tomiuk S, Hofmann K, Nix M, Zumbansen M, Stoffel W (1998). Cloned mammalian neutral sphingomyelinase: functions in sphingolipid signaling?. *Proceedings of the National Academy of Sciences of the United States of America*.

[35] Diamond LS, Harlow DR, Cunnick CC (1978). A new medium for the axenic cultivation of *Entamoeba histolytica* and other Entamoeba. *Transactions of the Royal Society of Tropical Medicine and Hygiene*.

[36] Castro-Garza J, Anaya-Velazquez F, Said-Fernandez S, Gonzalez-Garza MT (1996). Comparable growth of a *Trichomonas vaginalis* strain in PEHPS and TYI-S-33 media. *Archives of Medical Research*.

[37] Said-Fernandez S, Vargas-Villarreal J, Castro-Garza J (1988). PEHPS medium: an alternative for axenic cultivation of *Entamoeba histolytica* and *E. invadens*. *Transactions of the Royal Society of Tropical Medicine and Hygiene*.

[38] Vargas-Villarreal J, Mata-Cárdenas BD, Deslauriers M (2003). Identification of acidic, alkaline, and neutral sphingomyelinase activities in Mycobacterium tuberculosis. *Medical Science Monitor*.

[39] Skispky JP, Barclay M, Lowestein JM (1969). Thin-layer chromatography of lipids. *Methods in Enzymology*.

[40] Barret AJ (1980). The classification of proteinases. *Journal of Ciba Foundation Symposium*.

[41] Beynon RJ (1988). Prevention of unwanted proteolysis. *New Protein Techniques*.

[42] Lowry OH, Rosebrough NJ, Farr AL, Randal RJ (1951). Protein measurement with the Folin phenol reagent. *The Journal of Biological Chemistry*.

[43] Boolootian RA, Boolootian RA (1966). Reproductive physiology. *Physyology of Ehinodermata*.

[44] More D, Nugent J, Hagan L (2004). Identification of allergens in the venom of the common striped scorpion. *Annals of Allergy, Asthma and Immunology*.

[45] Gomez-Marín JE, El’Btaouri H, Bonhomme A (2002). Involvement of secretory and cytosolic phospholipases a2 during infection of IHP1 human monecytic cells with *Toxoplasma gondii*. Effect of interferon *γ*. *Parasitology Research*.

[46] Vargas-Villarreal J, Martínez-Rodríguez HG, Castro-Garza J, Mata-Cárdenas BD, González-Garza MT, Said-Fernández S (1995). Identification of *Entamoeba histolytica* intracellular phospholipase A and lysophospholipase L1 activities. *Parasitology Research*.

[47] Mata-Cárdenas BD, Hernández-García ME, González-Salazar F (2012). Axenic cultivation and comparative phospholipase A_2_ activity of *Giardia duodenalis* in a serum-free medium. *Acta Parasitology*.

[48] Mirhaghani A, Warton A (1998). Involvement of *Trichomonas vaginalis* surface-associated glycoconjugates in the parasite/target cell interaction. A quantitative electron microscopy study. *Parasitology Research*.

[49] Arroyo R, Engbring J, Alderete JF (1992). Molecular basis of host epithelial cell recognition by *Trichomonas vaginalis*. *Molecular Microbiology*.

[50] Alvarez-Sánchez ME, Avila-González L, Becerril-García C, Fattel-Facenda LV, Ortega-López J, Arroyo R (2000). A novel cystein proteinase (CP65) of *Trichomonas vaginalis* involved in cytotoxicity. *Microbial Pathogenesis*.

[51] Acosta A, Rael ED, Maddux NL, Lieb CS (1994). Detection of alkaline phosphatase in venom by blotting methods. *Toxicon*.

[52] Wiltshire CJ, Sutherland SK, Fenner PJ, Young AR (2000). Optimization and preliminary characterization of venom isolated from 3 medically important jellyfish: the box (*Chironex fleckeri*), irukandji (*Carukia barnesi*), and blubber (*Catostylus mosaicus*) jellyfish. *Wilderness and Environmental Medicine*.

[53] Wikiel H, Zhao L, Gessner T, Bloch A (1994). Differential effect of growth- and differentiation-inducing factors on the release of eicosanoids and phospholipids from ML-1 human myeloblastic leukemia cells. *Biochimica et Biophysica Acta*.

[54] Silva LC, Futerman AH, Prieto M (2009). Lipid raft composition modulates sphingomyelinase activity and ceramide-induced membrane physical alterations. *Biophysical Journal*.

[55] Said-Fernandez S, Lopez-Revilla R (1982). Subcellular distribution and stability of the major hemolytic activity of *Entamoeba histolytica* trophozoites. *Zeitschrift fur Parasitenkunde*.

[56] Neale KA, Alderete JF (1990). Analysis of the proteinases of representative *Trichomonas vaginalis* isolates. *Infection and Immunity*.

